# Microbial Distribution and Abundance in the Digestive System of Five Shipworm Species (Bivalvia: Teredinidae)

**DOI:** 10.1371/journal.pone.0045309

**Published:** 2012-09-20

**Authors:** Meghan A. Betcher, Jennifer M. Fung, Andrew W. Han, Roberta O’Connor, Romell Seronay, Gisela P. Concepcion, Daniel L. Distel, Margo G. Haygood

**Affiliations:** 1 Division of Environmental and Biomolecular Systems, Oregon Health & Science University, Beaverton, Oregon, United States of America; 2 Ocean Genome Legacy, Ipswich, Massachusetts, United States of America; 3 Marine Science Institute, University of the Philippines, Diliman, Quezon City, Philippines; University of Chicago, United States of America

## Abstract

Marine bivalves of the family Teredinidae (shipworms) are voracious consumers of wood in marine environments. In several shipworm species, dense communities of intracellular bacterial endosymbionts have been observed within specialized cells (bacteriocytes) of the gills (ctenidia). These bacteria are proposed to contribute to digestion of wood by the host. While the microbes of shipworm gills have been studied extensively in several species, the abundance and distribution of microbes in the digestive system have not been adequately addressed. Here we use Fluorescence *In-Situ* Hybridization (FISH) and laser scanning confocal microscopy with 16S rRNA directed oligonucleotide probes targeting all domains, domains Bacteria and Archaea, and other taxonomic groups to examine the digestive microbiota of 17 specimens from 5 shipworm species (*Bankia setacea*, *Lyrodus pedicellatus*, *Lyrodus massa*, *Lyrodus* sp. and *Teredo* aff. *triangularis*). These data reveal that the caecum, a large sac-like appendage of the stomach that typically contains large quantities of wood particles and is considered the primary site of wood digestion, harbors only very sparse microbial populations. However, a significant number of bacterial cells were observed in fecal pellets within the intestines. These results suggest that due to low abundance, bacteria in the caecum may contribute little to lignocellulose degradation. In contrast, the comparatively high population density of bacteria in the intestine suggests a possible role for intestinal bacteria in the degradation of lignocellulose.

## Introduction

A variety of terrestrial and aquatic animals, including termites and related insects, and certain mammals, reptiles, fish and birds, ingest fibrous or woody lignocellulose-rich plant materials as food [1]. Each of these organisms harbor abundant and well-described microbial communities in their digestive systems thought to aid in the decomposition of recalcitrant plant cell wall materials (lignocellulose) and in the supplementation of their nutritionally imbalanced diets [1]–[5].

**Figure 1:**
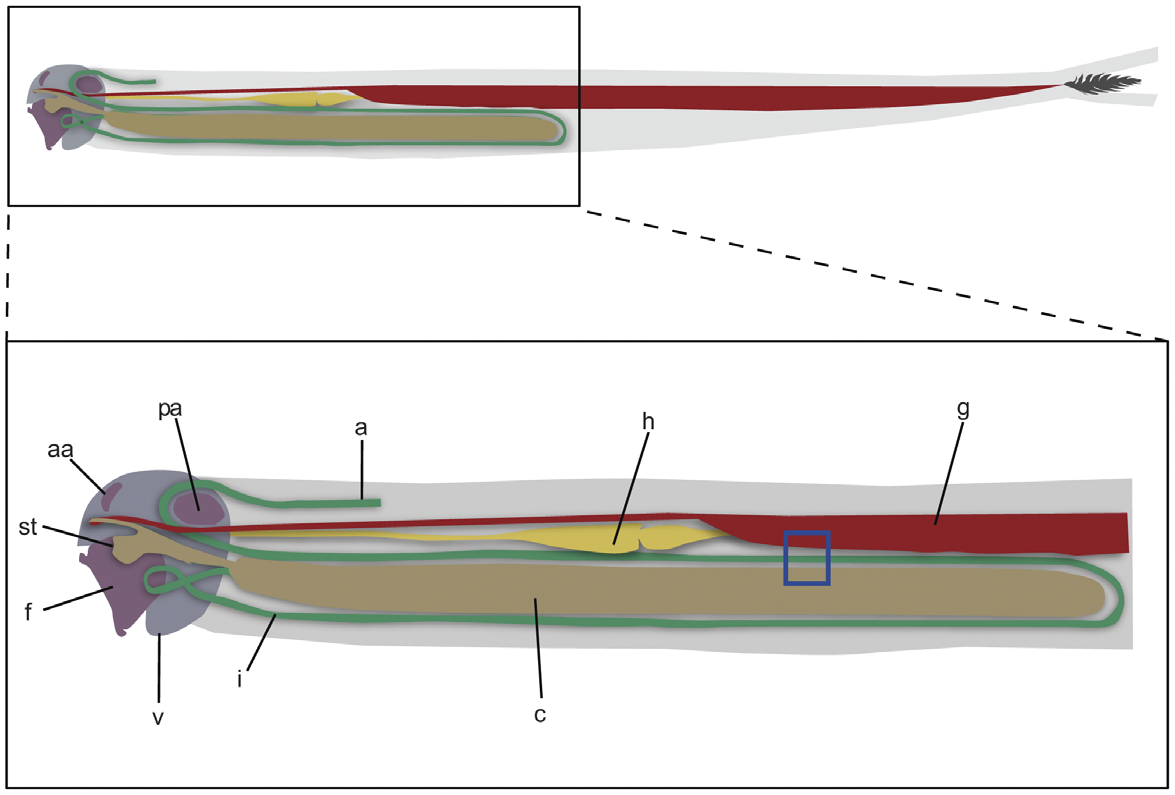
Anatomy of the shipworm *Bankia setacea*. a, anus; c, caecum; f, foot; g, gill; h, heart; i, intestine; v, valve (shell); aa, anterior adductor muscle; pa, posterior adductor muscle; st, stomach. Caecum and intestine are shown in brown and green respectively. Region boxed in blue corresponds to (A) in Figure 2.

In contrast to other xylophagous animals, little is known about the digestive microbiota of wood-feeding bivalves of the family Teredinidae (shipworms), the most important consumers of wood in many shallow (0–150 m) marine environments [6]. Most shipworm species possess a simple digestive system with a large caecum and a relatively short and straight intestine (Figure 1). Conspicuous microbial communities like those observed in the hindgut of various termite species, have not been reported in shipworms. Indeed, the few available reports suggest that shipworms and relatives may harbor few microbes in their digestive systems [7], [8]. In contrast, dense intracellular communities of microbes have been observed within specialized cells (bacteriocytes) of their gills (ctenidia) [9]–[12]. These endosymbionts have been shown to fix atmospheric nitrogen to forms usable by the host [13], and when grown *in vitro*, to produce cellulases (enzymes that depolymerize cellulose to shorter oligocellosaccharides) and other lignocellulose-active enzymes [14]–[16]. Thus, these symbionts have been proposed as a possible source of cellulolytic enzymes to aid in digestion of wood.

While the endosymbionts found in the gills of shipworms have been extensively characterized using micrographic and molecular methods [11], [12], no systematic examination of the abundance and distribution of microbes in the digestive system of shipworms have been attempted. Thus the potential role of microbial fermentation in the digestive system of shipworms remains largely unexplored. Here we use micrographic and hybridization histochemistry methods to explore the location and relative abundance of microbes in the caecum and intestine of five species and discuss the potential role of these microbes in lignocellulose degradation in these animals.

## Results

### Localization of Microbes in the Caecum and Intestine

An investigation including 191 tissue sections of 21 shipworm specimens covering 5 shipworm species was completed. Our efforts focused on the shipworm species Bankia setacea, from Oregon and Washington state, and Lyrodus pedicellatus, both from culture and wild specimens from Bohol Island, the Philippines with an analysis of 12 and 6 specimens respectively. To extend our study to additional shipworm species we included one specimen each of Lyrodus massa, Teredo aff. triangularis, and Lyrodus sp. from Bohol Island, the Philippines. These specimens originated from a diverse range of habitats and locations including, cold waters of the United States Pacific Northwest, warm equatorial mangrove and reef environments, and a captive population maintained in culture at Ocean Genome Legacy (Table 1).

**Table 1:** Shipworm specimens examined in this study.

Species	Collection Site	Number of specimens
*Bankia setacea*	Yaquina Bay OR, USA	2
	Puget Sound WA, USA	10
*Lyrodus pedicellatus*	Bohol, Philippines,	2
	OGL colony^1^	4
*Lyrodus massa*	Bohol, Philippines	1
*Lyrodus* sp.	Bohol, Philippines	1
*Teredo* aff. *triangularis*	Bohol, Philippines	1
	**Total**	21

1 captive breeding colony maintained at Ocean Genome Legacy, Ipswich MA, original collection location Long Beach, California.

Although the specimens observed covered a diverse selection of shipworm species and habitats, results of our examination were remarkably consistent across all samples. The caecae of 21 shipworms contained an abundance of woody material indicating active wood consumption [17] by the animal at the time of sacrifice. This study focused on shipworms actively consuming wood as evidenced by a full caecum and a burrow showing clean, solid and unobstructed wood at the excavation face. Thus specimens showing signs of decreased wood consumption (2 of 23 specimens examined) were excluded.

**Figure 2:**
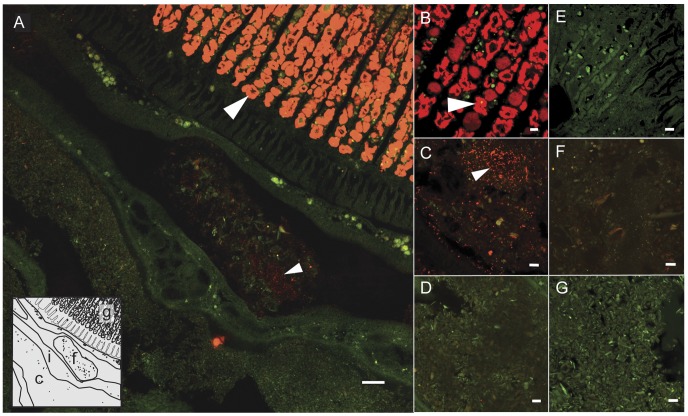
Single probe localization of bacteria in the gills and digestive system of *L. pedicellatus*. Confocal micrographs and diagrams demonstrating patterns of hybridization of either a bacteria-domain targeted 16S rRNA directed oligonucleotide probe (Bact338-cy5) or a negative control probe (Non338-cy5, to reveal autofluorescence or non-specific binding) to adjacent tissue sections of *L. pedicellatus* containing both gill and digestive tissues. Both probes are labeled with the fluorochrome cy5 (red). (A) Tissue section showing gill filaments (upper right), caecum (lower left) and intestine (diagonal across center, upper left to lower right) probed with Bact338-cy5. Inset- Diagram showing detail of tissue boundaries in the tissue section shown in (A). This region corresponds to the blue box in Figure 1. (B–G) Enlarged views of tissue sections hybridized with bacteria specific probe (Bact338-cy5; B–D) and negative control probe (Non338-cy5; E–G). These sections contain: gill bacteriocytes, (B,E), a fecal pellet within intestine (C,F), and caecum contents (D,G). Note that fluorescence signal localizes bacteria in gill bacteriocytes (large arrows) and in fecal pellets (small arrows), but few bacteria are observed in caecum contents. c, caecum; f, fecal pellet; g, gill; i, lumen of intestine. Scale bar in (a), 50 µm; (B–G), 10 µm.

**Figure 3:**
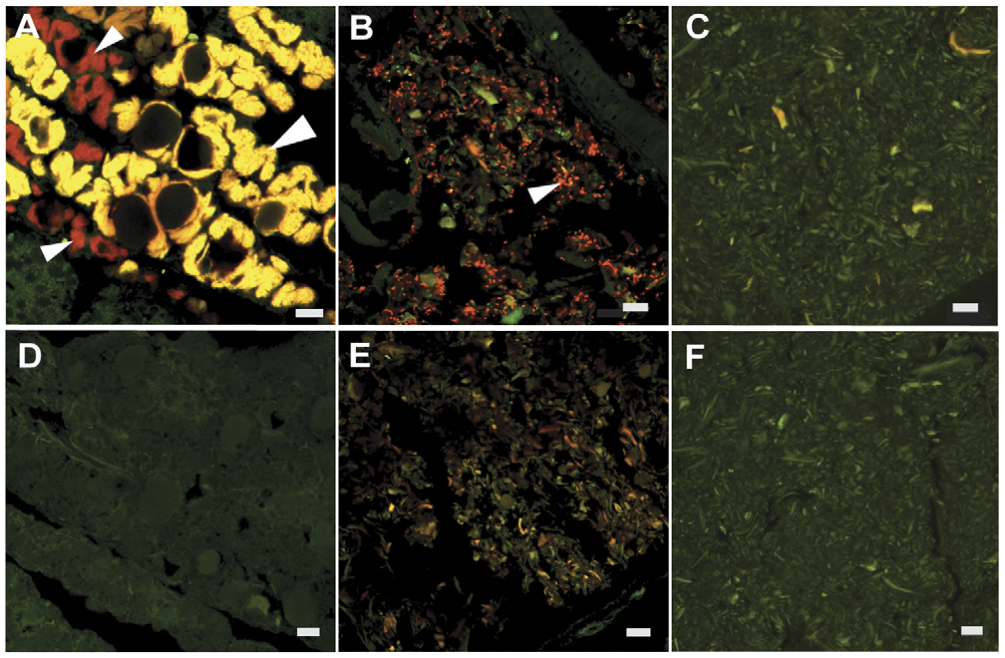
Dual probe localization of bacteria in the gills and digestive system of *L. massa*. Confocal micrographs demonstrating patterns of hybridization of 16S rRNA directed oligonucleotide probes targeted to the domain bacteria (Bact338-cy5, red) and shipworm symbionts (SS1273-cy3, green) and negative control probes (Non338-cy5, red and SSnon-cy3, green to reveal autofluorescence or non-specific binding). Micrographs show tissue sections hybridized with Bact338-cy5 and SS1273-cy3 (A–C) or Non338-cy5 and SSnon-cy3 (D–F). These sections contain: gill bacteriocytes (A, D, dark voids are host nuclei and lysosomal residual bodies), a fecal pellet within the intestine (B,E), and caecum content (C,F). The combinatorial color yellow indicates hybridization of both bacteria- and symbiont-targeted probes within the same bacterial cells or bacteriocytes. The appearance of both red bacteriocytes (small arrows) and yellow bacteriocytes (large arrows) in (A) indicates that bacteria within some bacteriocytes contain the symbiont sequence targeted by probe SS1273 while others contain bacteria that lack this target sequence. Note that hybridization of the symbiont-targeted probe SS1273 was detected in gill bacteriocytes only, and not in other examined tissues. All scale bars are 10 µm.

**Figure 4:**
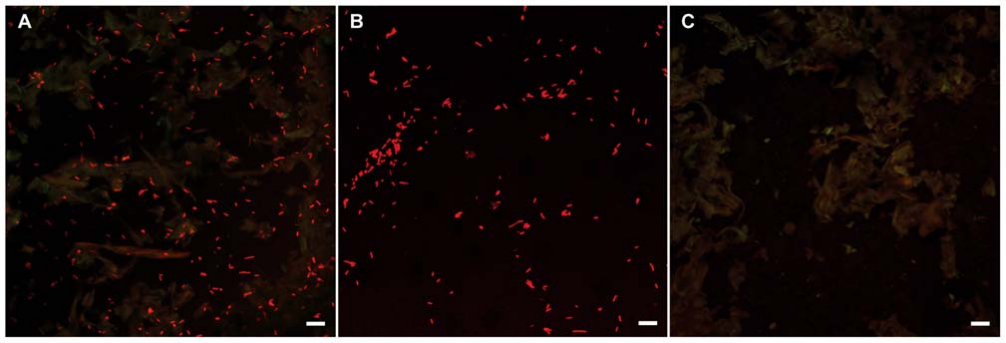
Single probe localization of exogenous bacteria added to caecum contents. Confocal micrographs depicting hybridization of a bacteria-domain specific 16S rRNA directed oligonucleotide probe (Bact338-cy5) to samples on filters. Probe is labeled with the fluorochrome cy5 and displayed in red in images shown. (A) Filter with a mixture of caecum contents and *E. coli* cells. (B) An equivalent sample of *E. coli* cells without caecum contents. (C) Equivalent sample of caecum contents without added *E. coli* cells. Scale bar 10 µm.

In contrast to shipworm gills [11], very few microbes were detected in the caecum (Figure 2, panels a and d). In approximately 60% of specimens a few bacteria, usually present in clusters of 3–6 cells, were detected within the cellulosic substrate. In approximately 70% of the cases in which bacteria were detected in the caecum, the bacterial cells were located in the anterior region of the caecum near the entrance to the stomach. No additional microbes were detected through hybridization with universal probe U1390, which targets bacteria, eukaryotes, and archaea. Dual probe hybridizations revealed that the few bacterial cells located in the caecum did not hybridize with the shipworm symbiont targeted probe, SS1273, (Fig. 3, panel c). This observation is consistent with previous results [11] in which endosymbiont ribotypes found in the gill were not detected in other tissues. A control experiment, in which caecum contents were spiked with bacteria prior to hybridization, demonstrated that caecum contents do not interfere with the method used for visualizing bacteria in this investigation (Figure 4).

**Figure 5:**
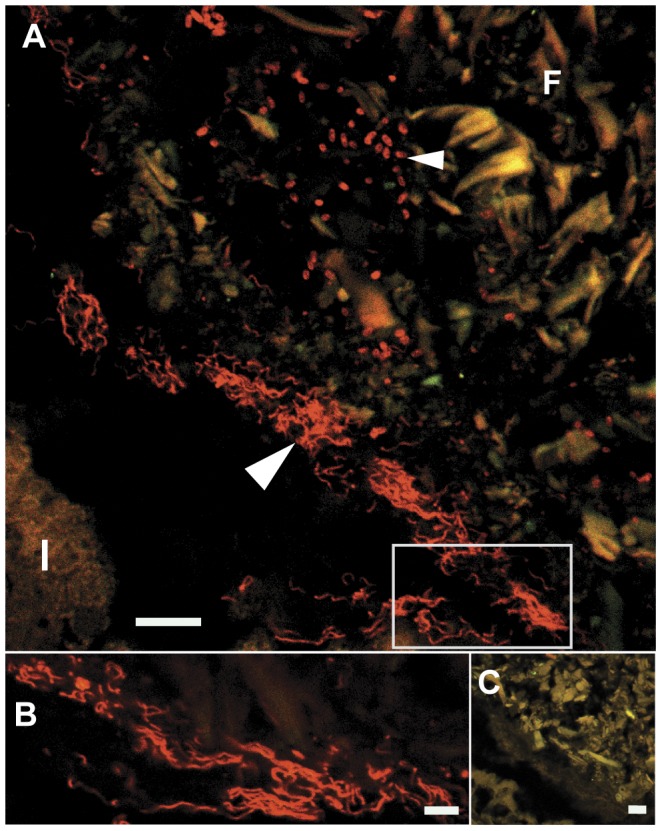
Bacterial morphotypes in the intestine of *Bankia setacea* visualized by single probe hybridization. Confocal micrographs depicting hybridization of either a bacteria-domain specific 16S rRNA directed oligonucleotide probe (Bact338-cy5) or a negative control probe (Non338-cy5) to sectioned tissues of *Bankia setacea*. Both probes are labeled with the fluorochrome cy5 shown in red. (A) Tissue section hybridized with Bact338-cy5, showing a fecal pellet (F) with a small portion of the intestinal wall (I) visible in the lower left corner. Note the presence of multiple cell morphologies including long spirals (large arrow) and short rods or cocci (small arrow). (B) Enlarged view of the boxed region in A. (C) Tissue section adjacent to and comparable in size to that shown in (A) hybridized with Non338-cy5. Scale bars are 10 µm in (A) and (C) and 5 µm in (B).

While the microbial density in the caecum was notably low, a morphologically diverse community of bacteria was observed in the intestines (Figure 5). Within the fecal pellets a comparatively dense community of cocci and rod-shaped bacteria was observed. In addition, spiral shaped bacteria were detected in the intestine of greater than half of all specimens analyzed (Figure 5b). Approximately 90% of spiral shaped bacteria detected were located on the outer surface of the fecal pellets, along the intestinal walls, and in the spaces between.

Detectable microbial populations were consistently observed in the intestines of all specimens. Variation was observed among individuals with respect to the density and appearance of microbial cells at various locations along the length of the intestine from anterior to posterior, however no consistent pattern was discerned. Hybridization of the shipworm symbiont targeted probe (SS1273) to microbial cells was detected only within the gills (Figure 3) and hybridization of the archaea-targeted probe (Arch 915) was not detected in any sections examined (not shown).

**Figure 6:**
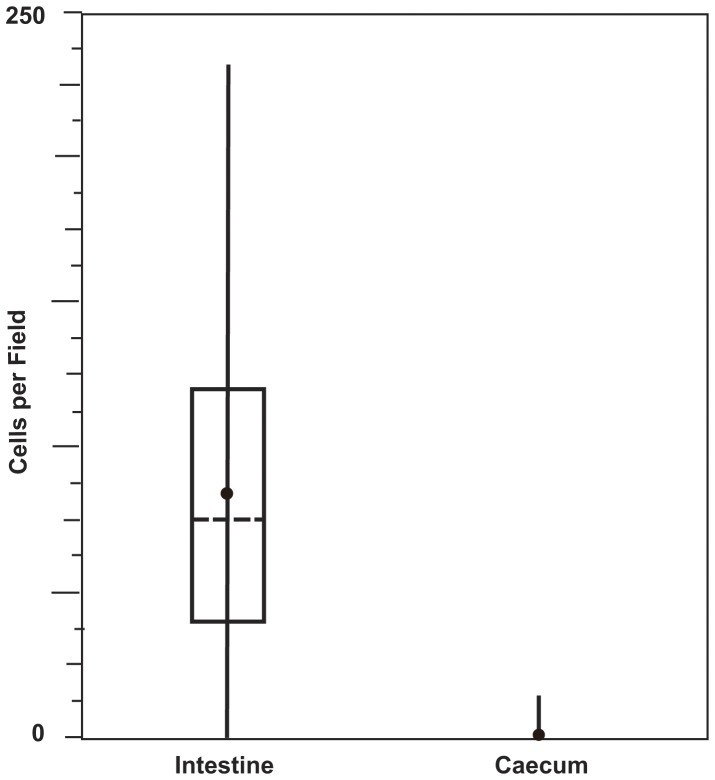
Abundance of bacteria observed in contents of intestine and caecum. Box plot showing number of bacteria observed per field in intestine and caecum of four shipworm species. Forty randomly selected fields (100 µm×100 µm) were examined for each tissue by laser scanning confocal microscopy using the bacteria-targeted probe Bact338 cy5 or the negative control probe Non338 cy5. Vertical lines indicate range of values, horizontal dashed lines and solid circles indicate median and mean values respectively, and open boxes indicate upper (top line) and lower (bottom line) quartiles respectively. Note upper and lower quartiles and median in caecum have zero values and have been omitted for clarity.

Cell counts, with background subtracted, in 40 fields (100 µm×100 µm) in caecum and intestine in 4 specimens revealed an average of 0.88 cells/field with a range of 0–14 cells/field and standard deviation of 2.8 cells/field in the caecum and an average of 83.8 cells/field with a range of 0–224 cells/field and standard deviation of 61.3 cells/field in the intestine. Results are shown in Figure 6.

## Discussion

With few exceptions, shipworms are obligate wood-borers that use wood as a source of shelter and that ingest large quantities of the excavated wood particles as they burrow [18], [19]. To date, at least 65 shipworm species have been described comprising 14 genera distributed broadly in temperate to tropical marine and brackish environments across the globe [20]. Due to their voracious appetite for woody substrates, shipworms are thought to play an important role in remineralization of plant matter, carbon cycling and destruction of wooden structures in marine environments [6]. Shipworms have also received attention recently as a potential source of enzymes for application in the conversion of lignocellulose to fermentable sugars, a major bottleneck in the production of liquid biofuels from cellulosic feedstocks [21]–[23]. Species of the subfamily Bankiinae and members of the *Lyrodus* lineage of the subfamily Terediniinae [21] have received the most research attention due to their wide geographic distribution and role in destruction of marine structures and are the focus of this investigation.

The mechanisms by which and extent to which shipworms digest woody plant material are not well understood. Although many shipworms are capable of filter feeding [19], [20] the comparatively small size of the labial palps has been interpreted as evidence of reduced reliance on this feeding method [20]. Indeed, *L. pedicellatus* has been shown to be capable of normal growth and reproduction with wood as its sole particulate food [19]. To explore the potential mechanism of lignocellulose degradation in *L. pedicellatus* and four other species, we have examined the distribution and abundance of microorganisms in two regions of the digestive system, the caecum and intestine.

Most animals that subsist predominantly on lignocellulose-rich plant materials rely, at least to some extent, on enzymes produced by microorganisms to reduce this insoluble composite material to soluble and more readily digested components. In the vast majority of cases, these microbes utilize fermentative metabolism and are located within anatomically distinct regions of the digestive system.

In shipworms, burrowing and particle size reduction are accomplished by mechanical abrasion using micron-scale tooth-like projections on the surfaces of the valves (shells). After ingestion, wood particles pass through the esophagus and stomach [24], which are proportionally small by comparison to those of other bivalves [20]. Wood particles then pass into the caecum or appendix, a large blind sac appended to the stomach, and accumulate there. This conspicuous organ is often orders of magnitude larger in volume than the stomach and may extend up to 60% of the body length [20]. The caecum is a diagnostic feature of wood feeding bivalves. After passage through the caecum, wood particles are returned to the stomach and passed to a relatively short, simple, tubular intestine [25]. Overview in Figure 1.

Based on anatomical and biochemical considerations, the caecum has been proposed to be a principal site of wood digestion and absorption in *Lyrodus*, *Bankia* and *Teredo*. For example, reducing sugar assays show evidence of the depolymerization of cellulose in caecal content but not in pre-caecal material in shipworms of the genus *Teredo* [7]. Furthermore, the internal surface area of the caecum is expanded by the presence of a large typhlosole, a y-shaped fold of the internal wall surface extending the full length of the caecum. The inner surface of the caecum, including the typhlosole, is lined by a brush border microvillar epithelium [17]. The resulting large internal surface area of the caecum and the presence of extensive vascularization and a significant caecal artery passing directly from the heart to the caecum [25] have been interpreted as evidence that the caecum is specialized for absorption of soluble end products of lignocellulose hydrolysis [17].

The results presented here indicate that a gut microbiota is evident in five shipworm species and that all five species show a consistent pattern of microbial density and distribution. In each case, an extremely sparse microbial community is observed in the caecum, while well-developed and morphologically varied populations of bacteria are observed within the intestines. These intestinal microbes are mainly confined to the interior and surfaces of discrete boluses of wood particles (fecal pellets) passing through the gut.

The phylogenetic composition of the microbial communities detected in the intestines of these shipworm species remains to be determined. However, results presented here indicate that these communities are distinct from those found in shipworm gills. Probes that were used successfully to detect gill endosymbiont ribotypes in the gill region of the same tissues sections did not hybridize with the microbes detected in the digestive system, indicating that the phylogenetic compositions of these two communities are distinct.

It is also evident from inspection that the densities of microbial populations in the digestive tract are far lower than those of the gill bacteriocytes and the volume of the intestine is considerably smaller than that of the gill tissue. This suggests that the total biomass of bacterial cells in the gills significantly exceeds that of the digestive system.

The absence of a developed microbial community in the caecum of these shipworm species suggests a digestive mechanism distinct from those observed in many other cellulotrophic symbioses. For example, in lower termites, protozoan flagellates in the gut are responsible for lignocellulose degradation, which results in the release of acetate, CO_2_, and H_2_. This is followed by anaerobic fermentation of by-products by bacterial methanogens and acetogens [5], [26]. Similarly, in the cow rumen a consortium of bacteria, protozoa, and fungi digest lignocellulose, providing acids, sugars, and gases to a fermentative bacterial community. In both of these systems the host receives nutriment from volatile fatty acids, end products of microbial symbiont metabolism [3], [26].

Enzymes encoded by the host nuclear genome may also contribute to lignocellulose degradation. For example, host derived enzymes, including cellulases, xylanases and cellobiohydrolases have been identified in termites, wood eating roaches, arthropods, mollusks, and nematodes [27]–[29]. Higher termites utilize both microbially produced cellulases and enzymes produced by the host in lignocellulose degradation [5], [30]. In the case of the wood boring marine isopod *Limnoria quadripunctata* the digestive system appears to lack a significant microbial community and is thought to rely on host-encoded cellulases for lignocellulose digestion [31].

The low abundance of microbial prokaryotes and eukaryotes in the caecum, the largest part of the shipworm digestive system and a demonstrated site of lignocellulose breakdown [7], suggests that contact dependent microbial lignocellulose degrading activity and microbial fermentative metabolism by local microbes may play a comparatively small role in this symbiosis. If this is the case, the cellulolytic activity observed in shipworm caecum may be due to endogenous cellulases encoded in the nuclear genome of the host. Alternatively, cellulolytic enzymes produced remotely by gill endosymbionts could contribute to this hydrolytic activity, although this has not been demonstrated and no precedent exists for such a mechanism in intracellular endosymbionts. In either case, investigation of the origin, nature and locations of cellulase enzymes in the shipworm system will be important.

The intestinal anatomy of the shipworm species examined here does not reveal features typical of organisms that rely heavily on fermentation. The intestines are comparatively short, straight, low in volume and cross-sectional area, and have no obvious convolutions, chambers, caeca, folds, or haustrations. Nonetheless, the presence of a conspicuous microbial community in the intestines suggests a possible role for intestinal bacteria in wood digestion.

The marked difference between the microbial abundance in the caecum and intestine is also intriguing. In the mammalian digestive system, ingested material passes first through the stomach, where microbial abundance is low, and then to the intestine where microbial abundance increases markedly due to differences in the conditions and residence time. Similarly, conditions must also differ sufficiently in the caecum and intestine of shipworms to account for the different microbial communities.

The conditions that explain this difference in microbial abundance are unknown. Due to the detected hydrolytic activity in the caecum, this location is expected to be rich in soluble and insoluble carbohydrates, oligosaccharides and sugars and so might be expected to support a local microbial population. Such microbial growth could compete with the host for breakdown products of wood and so mechanisms that might prevent such growth could provide a growth advantage to the host and so deserve investigation.

In conclusion, results of this study confirm the absence of a substantial microbial community in the caecum of five shipworm species, and reveal an as yet un-characterized and morphologically diverse microbial community in the intestines. This provides rationale for further exploration of the role of the intestinal microbial community in lignocellulose degradation and presents further knowledge regarding the basic understanding of xylotrophy in this unique and biotechnologically relevant symbiosis.

## Materials and Methods

### Collection of Shipworm Specimens

*B. setacea* specimens were collected from Yaquina Bay, OR (44.6°N, 124.0°W) and Puget Sound, WA (47.9°N, 122.4°W) in found wood and in pine collection panels deployed at depths ranging from 1–3 m for 6–9 months during July 2009 to December 2010. After collection, specimens and their wood substrates were maintained in seawater aquaria at 10°C without addition of other food sources. Laboratory grown specimens of *L. pedicellatus* were obtained from a captive breeding population derived from specimens originally collected from Long Beach, CA and currently maintained at Ocean Genome Legacy, Ipswich, MA, Wild specimens of *L. pedicellatus*, *L. massa*, *Lyrodus* sp. and *Teredo* aff. *triangularis* were collected in Bohol Island, the Philippines (10°N, 124°E), from pine and yellow lauan collection panels deployed in mangroves or reefs at depths ranging from 1 m to 17 m for 9 months from September 2009 to January 2011.

Prior to fixation, specimens were extracted from the wood by hand, rinsed in sterile seawater and either fixed whole or dissected to remove individual tissues of interest. Individual tissues were washed thoroughly in sterile seawater prior to fixation. See Table 1 for a list of specimens included in this analysis and Table S1 for more detail on the specimens included in this study.

### Specimen Fixation and Histological Preparation

Samples for microscopy were fixed in 4% paraformaldehyde in phosphate buffered saline (PBS, 10 mM Na2HPO4, 2.7 mM KCl, 140 mM NaCl, pH 7.4) at 4°C overnight then transferred to 70% ethanol and stored at −20°C.

Samples for fluorescence *in situ* hybridizations (whole animal or individual tissue) were processed in an automatic tissue processor (Tissue Tek-VIP Processor), embedded in paraffin (Paraplast or Tissue Prep 2, melting temperature 56°C), sectioned to either 5 or 10 µm thickness on a Micron rotary microtome, and mounted on glass slides (Superfrost Plus, Fisher Scientific) at the OHSU Oregon National Primate Research Center Histology Core facility (Beaverton, OR) or Beth Israel Deaconess Medical Center Histology Lab (Boston Massachusetts). A subset of sections from each sample was stained with haematoxylin and eosin to facilitate identification of tissues of interest. To provide for the most complete analysis of specimens, cross-sections and sagittal sections were included in this study.

### Control Experiments with Caecum Contents

Caecum contents were collected from 8 wood feeding specimens of *L. pedicellatus* from the Ocean Genome Legacy colony, combined and stored at −20°C. *Escherichia coli* cells were grown overnight, then sedimented by centrifugation and resuspended in 1/10 volume of filtered seawater. Three samples were prepared for comparison: 20 µl of caecum contents plus 5 µl of E. coli, 20 µl of filtered seawater plus 5 µl of E. coli, and 20 µl of caecum contents. The samples were fixed in 4% paraformaldehyde in seawater overnight at 4°C, then transfered into 70% ethanol. Each sample was filtered onto a 0.2 um pore size black polycarbonate filter (Osmonics), and hybridized and visualized as below.

### Localization of Bacteria by Fluorescence *in situ* Hybridization

Prior to hybridization, paraffin was removed from sections by incubation in xylenes (5 minutes) followed by 2 rinses, one minute each, in 95% ethanol and water filtered by a Milli-Q system (Millipore, New Bedford, MA). Oligonucleotide probe hybridizations were performed on slide-mounted tissue sections as in (Sharp et al. 2007). Bact338, a Domain Bacteria-targeted 16S rRNA probe [32], was used to survey the tissues for bacteria. A second probe, U1390, targeting a 16S rRNA sequence conserved in all domains [33], was used in selected hybridizations to achieve broader specificity of detection of microorganisms. The general archaeal probe Arch 915 [34] was implemented to detect the presence of archaea.

ARB Probe Design tool [35] was used to design probe SS1273, which is complementary to a short region conserved in the 16S rRNA sequences previously reported for shipworm endosymbiont ribotypes but that differs from those of most other bacteria and archaea. Sequences used in the design of SS1273 are listed in Table S2. Probe design parameters: length of probe 21, temperature 40.0–63.0, GC content 30.0–70.0, E.coli position 1–100000, maximum non group hits 2, minimum group hits 90%, SILVA v. 100.

Comparison to sequences in the Ribosomal Database Project sequence database [36](http://rdp.cme.msu.edu/index.jsp) indicates potential cross-hybridization with 5 non-target taxa including three members of the Deltaproteobacteria and two Betaproteobacteria). The SS1273 probe hybridized to fixed cells from pure cultures of the known shipworm symbiont *Teredinibacter turnerae* T7901, but not to the marine CFB bacterium *Microscilla marina* ATCC 23134, supporting the specificity of the probe for gill endosymbionts. Probes Non338, the reverse complement of the Bact338 sequence, and SSNon, containing one internal base pair mismatch (G to T at position 1284) to SS1273, were used as negative controls in all experiments. Probes were labeled at the 5′ end with either the Cy3 or Cy5 fluorophore and were synthesized commercially (Integrated DNA Technologies, San Diego, CA). See Table 2 for probes used in this study. Probes were added to a final concentration of 5 ng/µL. Hybridization and washing procedures detailed in Sharp et al. 2007 were followed. Slides were mounted in 4∶1 Citifluor (Citifluor Ltd. London, UK): Vectashield (Vector Labs, Burlingame, CA, USA) prior to visualization.

**Table 2:** Fluorescence labeled oligonucleotide probes used in Fluorescence *in situ* hybridizations.

Probe	Sequence	Target	Reference
Bact338	GCTGCCTCCCGTAGGAGT	Universal Bacteria	Amann et al. 1990
NON338	ACTCCTACGGGAGGCAGC	NA	Manz et al. 1992
SS1273	ACTGTTTTATGGGATTAGCTC	Shipworm Symbionts	This Study
SSNon	ACTGTTTTATGTGATTAGCTC	NA	This Study
Arch915	GTGCTCCCCCGCCAATTCCT	Universal Archaea	Stahl and Amann 1991
U1390	GACGGGCGGTGTGTACAA	All Domains	Zheng et al. 1996

A Zeiss Axio Imager M1 laser scanning confocal microscope with probe-appropriate excitation wavelengths and LSM 5 Pascal Version 4.0 image-acquisition software (Carl Zeiss, Oberkerchen, Germany) were used for visualization and image capture. All imaging settings were held constant during capture of positive and corresponding negative control samples. Hybridizations and subsequent visualizations were carried out at Oregon Health & Science University and Ocean Genome Legacy by two authors (MAB & JMF) independently.

To quantify the results, images of sectioned caecum and intestine from 4 specimens (2 *L. pedicellatus*, 1 *L. massa*, and 1 *Lyrodus* species) that had been treated with the bacteria-targeted probe Bact338 cy5 were chosen at random. Fluorescent particles were counted manually for 10 fields of 100 µm×100 µm in each specimen. Fluorescent particles were similarly counted for 40 fields of 100 µm×100 µm in paired adjacent sections that had been treated with a negative control probe Non338 cy5. The mean number of fluorescent particles per field observed in the caecum using the negative control probe was subtracted from values observed using the experimental probe in the caecum and the mean number of fluorescent particles per field observed in the intestine using the negative control probe was subtracted from values observed using the experimental probe in the intestine. Counts were done using unaltered images. Brightness and contrast were adjusted to prepare figures, with identical adjustments applied to corresponding negative control images.

## Supporting Information

Table S1 Shipworm specimens used in this study. Expanded version of Table 1.
(DOC)

Table S2 Sequences used in the design of probe SS1273. The probe SS1273 was designed to be complementary to the 16S rRNA gene sequence of these publicly available strains.
(DOC)
