# Supplementary material for: Microbial Distribution and Abundance in the Digestive System of Five Shipworm Species (Bivalvia: Teredinidae)
Source: PLoS One. 2012 Sep 20;7(9):e45309. doi: 10.1371/journal.pone.0045309 (PMC3447940; doi:10.1371/journal.pone.0045309)
Supplement: Table S1 — Shipworm specimens used in this study. Expanded version of Table 1. (DOC) [file pone.0045309.s001.doc]

**Table S1. Shipworm specimens used in this study.**

| **Specimen ID** | **Sample ID ^1^** | **Species** | **Collection Site** | **Location** |
| --- | --- | --- | --- | --- |
| OR Bs4 | NA | *Bankia setacea* | Yaquina Bay, OR | 44.6° N, 124.0° W |
| OR Bs5 | NA | *Bankia setacea* | Yaquina Bay, OR | 44.6° N, 124.0° W |
| OGL Bs1 | NA | *Bankia setacea* | Puget Sound, WA | 47.9°N, 122.4°W |
| OGL Bs2 | NA | *Bankia setacea* | Puget Sound, WA | 47.9°N, 122.4°W |
| BS1 | NA | *Bankia setacea* | Puget Sound, WA | 47.9°N, 122.4°W |
| BS2 | NA | *Bankia setacea* | Puget Sound, WA | 47.9°N, 122.4°W |
| BS3 | NA | *Bankia setacea* | Puget Sound, WA | 47.9°N, 122.4°W |
| BS4 | NA | *Bankia setacea* | Puget Sound, WA | 47.9°N, 122.4°W |
| BS5 | NA | *Bankia setacea* | Puget Sound, WA | 47.9°N, 122.4°W |
| BS9 | NA | *Bankia setacea* | Puget Sound, WA | 47.9°N, 122.4°W |
| 2 | NA | *Bankia setacea* | Puget Sound, WA | 47.9°N, 122.4°W |
| Bs Caecum | NA | *Bankia setacea* | Puget Sound, WA | 47.9°N, 122.4°W |
| LP1 | NA | *Lyrodus pedicellatus* | OGL Colony | 47.9°N, 122.4°W |
| OGL Lp2 | NA | *Lyrodus pedicellatus* | OGL Colony | 47.9°N, 122.4°W |
| PMS 1140T | PMS 1140U | *Lyrodus pedicellatus* | Philippines, Bohol, Bil-isan | 9.6° N, 123.7° E |
| PMS 1592U | PMS 1593W ^2^ | *Lyrodus pedicellatus* | Philippines, Bohol, Balicasag | 9.5° N, 123.7° E |
| PMS 785M | PMS 1180L | *Lyrodus massa* | Philippines, Bohol, Danajon Bank | 10.3° N, 124.4° E |
| PMS 1173T | PMS 1171P | *Lyrodus* sp*.* | Philippines, Bohol, Danajon Bank | 10.3° N, 124.4° E |
| PMS 1035H | PMS 1038M | *Teredo* aff*. triangularis* | Philippines, Bohol, Danao | 9.6° N, 123.7° E |

^1^Only applies to specimens collected by the Philippine Mollusk Symbiont- International Cooperative Biodiversity Group. ^2^ Three *L. pedicellatus* specimens were combined in this sample.
