# Supplementary material for: Microbial Distribution and Abundance in the Digestive System of Five Shipworm Species (Bivalvia: Teredinidae)
Source: PLoS One. 2012 Sep 20;7(9):e45309. doi: 10.1371/journal.pone.0045309 (PMC3447940; doi:10.1371/journal.pone.0045309)
Supplement: Table S2 — Sequences used in the design of probe SS1273. The probe SS1273 was designed to be complementary to the 16S rRNA gene sequence of these publicly available strains. (DOC) [file pone.0045309.s002.doc]

**Table S2. Sequences used in the design of probe SS1273.** The probe SS1273 was designed to be complementary to the 16S rRNA gene sequence of these publicly available strains.

| **Strain Name** | **Accession Number** | **Reference** |
| --- | --- | --- |
| *Teredinibacter turnerae* T7902 | AY028398 | Distel et al. 2002b |
| *Teredinibacter turnerae* CS30 | AY949835.1 | Distel et al. 2002b |
| *Teredinibacter turnerae* T8602 | EU604077.1 | Yang et al. unpublished |
| *Teredinibacter turnerae* T7901 | EU604078.1 | Yang et al. 2009 |
| *Teredinibacter turnerae* T0609 | EU604079.1 | Yang et al. unpublished |
| *Teredinibacter turnerae* CS32 | AY949836.1 | Trindade-Silva et al. 2009 |
| RT1 *L. pedicellatus* clone | DQ272300 | Luyten et al. 2006 |
| RT2 *L. pedicellatus* clone | DQ272301 | Luyten et al. 2006 |
| RT3 *L. pedicellatus* clone | DQ272302 | Luyten et al. 2006 |
| RT5 *L. pedicellatus* clone | DQ272303 | Luyten et al. 2006 |
| RT6 *L. pedicellatus* clone | DQ272304 | Luyten et al. 2006 |
| RT7 *L. pedicellatus* clone | DQ272305 | Luyten et al. 2006 |
| RT9 *L. pedicellatus* clone | DQ272306 | Luyten et al. 2006 |
| RT12 *L. pedicellatus* clone | DQ272307 | Luyten et al. 2006 |
| RT14 *L. pedicellatus* clone | DQ272308 | Luyten et al. 2006 |
| RT15 *L. pedicellatus* clone | DQ272309 | Luyten et al. 2006 |
| RT16 *L. pedicellatus* clone | DQ272310 | Luyten et al. 2006 |
| RT17 *L. pedicellatus* clone | DQ272311 | Luyten et al. 2006 |
| RT18 *L. pedicellatus* clone | DQ272312 | Luyten et al. 2006 |
| RT19 *L. pedicellatus* clone | DQ272313 | Luyten et al. 2006 |
| RT20 *L. pedicellatus* clone | DQ272314 | Luyten et al. 2006 |
| RT21 *L. pedicellatus* clone | DQ272315 | Luyten et al. 2006 |
| RT22 *L. pedicellatus* clone | DQ272316 | Luyten et al. 2006 |
| RT24 *L. pedicellatus* clone | DQ272317 | Luyten et al. 2006 |
| LP1 *L. pedicellatus* clone | AY150183 | Distel et al. 2002a |
| LP2 *L. pedicellatus* clone | AY150184 | Distel et al. 2002a |
| LP3 *L. pedicellatus* clone | AY150185 | Distel et al. 2002a |
| LP4 *L. pedicellatus* clone | AY150186 | Distel et al. 2002a |
| *B. setacea* clone | AF102866 | Sipe et al. 2000 |
